# Complement Component C1q as Serum Biomarker to Detect Active Tuberculosis

**DOI:** 10.3389/fimmu.2018.02427

**Published:** 2018-10-23

**Authors:** Rosalie Lubbers, Jayne S. Sutherland, Delia Goletti, Roelof A. de Paus, Coline H. M. van Moorsel, Marcel Veltkamp, Stefan M. T. Vestjens, Willem J. W. Bos, Linda Petrone, Franca Del Nonno, Ingeborg M. Bajema, Karin Dijkman, Frank A. W. Verreck, Gerhard Walzl, Kyra A. Gelderman, Geert H. Groeneveld, Annemieke Geluk, Tom H. M. Ottenhoff, Simone A. Joosten, Leendert A. Trouw

**Affiliations:** ^1^Department of Rheumatology, Leiden University Medical Center, Leiden, Netherlands; ^2^Medical Research Council Unit The Gambia at the London School of Hygiene and Tropical Medicine, Banjul, Gambia; ^3^Translational Research Unit, Department of Epidemiology and Preclinical Research, National Institute for Infectious Diseases, Rome, Italy; ^4^Department of Infectious Diseases, Leiden University Medical Center, Leiden, Netherlands; ^5^Department of Pulmonology, St. Antonius Hospital Nieuwegein, Nieuwegein, Netherlands; ^6^Department of Internal Medicine, St. Antonius Hospital Nieuwegein, Nieuwegein, Netherlands; ^7^Department of Nephrology, Leiden University Medical Center, Leiden, Netherlands; ^8^Pathology Service, National Institute for Infectious Diseases, Rome, Italy; ^9^Department of Pathology, Leiden University Medical Center, Leiden, Netherlands; ^10^Section of TB Research & Immunology, Biomedical Primate Research Centre, Rijswijk, Netherlands; ^11^Division of Molecular Biology and Human Genetics, Faculty of Medicine and Health Sciences, DST/NRF Centre of Excellence for Biomedical Tuberculosis Research, South African Medical Research Council Centre for Tuberculosis Research, Stellenbosch University, Cape Town, South Africa; ^12^Sanquin Diagnostic Services, Amsterdam, Netherlands; ^13^Department of Immunohematology and Blood Transfusion, Leiden University Medical Center, Leiden, Netherlands

**Keywords:** complement, tuberculosis, C1q, infection, innate immunity, blood, mycobacterium

## Abstract

**Background:** Tuberculosis (TB) remains a major threat to global health. Currently, diagnosis of active TB is hampered by the lack of specific biomarkers that discriminate active TB disease from other (lung) diseases or latent TB infection (LTBI). Integrated human gene expression results have shown that genes encoding complement components, in particular different C1q chains, were expressed at higher levels in active TB compared to LTBI.

**Methods:** C1q protein levels were determined using ELISA in sera from patients, from geographically distinct populations, with active TB, LTBI as well as disease controls.

**Results:** Serum levels of C1q were increased in active TB compared to LTBI in four independent cohorts with an AUC of 0.77 [0.70; 0.83]. After 6 months of TB treatment, levels of C1q were similar to those of endemic controls, indicating an association with disease rather than individual genetic predisposition. Importantly, C1q levels in sera of TB patients were significantly higher as compared to patients with sarcoidosis or pneumonia, clinically important differential diagnoses. Moreover, exposure to other mycobacteria, such as *Mycobacterium leprae* (leprosy patients) or BCG (vaccinees) did not result in elevated levels of serum C1q. In agreement with the human data, in non-human primates challenged with *Mycobacterium tuberculosis*, increased serum C1q levels were detected in animals that developed progressive disease, not in those that controlled the infection.

**Conclusions:** In summary, C1q levels are elevated in patients with active TB compared to LTBI in four independent cohorts. Furthermore, C1q levels from patients with TB were also elevated compared to patients with sarcoidosis, leprosy and pneumonia. Additionally, also in NHP we observed increased C1q levels in animals with active progressive TB, both in serum and in broncho-alveolar lavage. Therefore, we propose that the addition of C1q to current biomarker panels may provide added value in the diagnosis of active TB.

## Introduction

Tuberculosis (TB) is a major global health threat, which is caused by infection by *Mycobacterium tuberculosis* (*M.tb*) (1). Current estimations indicate that a quarter of the global population is infected with *M.tb*, with a life-long risk to develop active TB disease. Particular regions, such as South-East Asia, Western-Pacific, and Africa regions account for more than 80% of infected individuals (2). Annually over 6 million people are diagnosed with TB disease, a serious and highly contagious condition, resulting in 1.3 million deaths in 2016 only (1). While most infected individuals remain asymptomatic latently infected (LTBI), a minority (5–10%) of these individuals progress to active TB. Given the high rate of infections with *M.tb* in some regions it is important to discriminate infection from disease, which is difficult with the currently available tests. At present, only *M.tb* detection in sputum using smear, PCR or culture is definitive proof of TB disease. Early diagnosis and treatment of TB disease is important to reduce transmission of infection and prevent disease associated mortality (1).

Diagnosis of active TB is made by microbiological or genetic detection of *M.tb* in sputum (or other specimens in case of extrapulmonary TB), but this can be expensive and time-consuming depending on bacterial burdens or requires complex methodology and infrastructure. Current immunological tests can detect infection with *M.tb* but often fail to discriminate active disease from latent infection (3). Therefore, there is an urgent need to identify biomarkers that can discriminate active and latent TB infection in order to promptly initiate treatment to prevent mortality and further spread of the pathogen, in particular in areas where *M.tb* is highly endemic. Ideally, such biomarkers should also be able to discriminate between TB and other respiratory infections that present with similar symptoms and abnormalities on chest X-rays.

Many studies have identified potential biomarkers that discriminate active TB from LTBI, or that are predictive of which individuals will progress to active TB (4–10). Differential gene expression profiles between patients with TB and LTBI or other (lung) diseases resulted in identification of an array of potential biomarkers, such as *FCGR1A* (11–13) and *GBP5* (5, 6, 14). Recently, complement has been highlighted as candidate biomarker for active TB disease (15–19) also in the presence of HIV co-infection (20). Most currently identified biomarkers have been identified at the transcriptomic level; however, easy, robust markers that can be measured at the protein level would be more ideal candidates for application in the field. Therefore, validation of markers previously identified at the mRNA level at the protein level would provide important insights into the applicability of such markers in clinical practice.

Next to biomarker studies in humans, there are various experimental models to study the host-pathogen interaction. The best available model is the non-human primate model (NHP), infection of rhesus macaques with *M.tb*, resulted in TB disease which closely resembles human TB as they experience similar lesions and clinical courses as humans, suggesting a common pathophysiology (21). The NHP model adds important information on kinetics of disease development following *M.tb* infection and can be manipulated with e.g., different dosages of infection, different infecting strains, but also with vaccines.

The complement system is an important part of the innate immune system and functions as a proteolytic cascade. The classical complement pathway is initiated by binding of C1q to ligands, such as immune complexes. Following the binding of C1q to ligands, enzymatic processes lead to the release of inflammation stimulating peptides, C3a and C5a, formation of the opsonin C3b and formation of the membrane attack complex, resulting in target cell lysis (22). Furthermore, C1q can bind several receptors that contribute to other important functions, such as phagocytosis or myeloid cell modulation, outside traditional complement system activation (23, 24). For instance, C1q is involved in neovascularization during pregnancy, coagulation processes and neurological synapse function (25). C1q is a 480 kDa protein composed of six arms, each comprising one A, B and C peptide chain (26). These three chains are encoded by individual genes, *C1QA, C1QB*, and *C1QC*, located on chromosome 1p. In contrast to most complement proteins, C1q is not produced by hepatocytes but mainly by monocyte derived cells, such as macrophages and immature dendritic cells (27–29) and by mast cells (30). Increased expression of mRNA for C1q has been associated with TB disease (16, 19).

Here, we analyzed differential expression of complement genes in patients with TB. Since in publicly available datasets, C1q expression was most pronouncedly upregulated, we validated C1q at the protein level in samples from various patient groups as a biomarker for active TB. Patients with active TB disease were compared to latently infected individuals, vaccinnees and to patients with clinical conditions that are important differential diagnoses in clinical practice. Finally, to obtain more insight in the pathophysiology, kinetic analyses were performed samples obtained from NHP animal models of TB.

## Materials and methods

### Patients and controls

Demographic data and classification of the cohorts are presented in Table 1. Below we have specified the specific inclusion criteria per cohort.

**Table 1:** Description of the cohorts.

**Country**	**Classification**	***N***	**Age mean (range)**	**Sex (%male)**
Italy	Control	15	38 (25–57)	40
	Latent TB	18	37 (21–77)	33
	Active TB	18	38 (23–67)	89
	Treated TB	17	39 (18–70)	35
The Gambia	Control	50	31 (15–60)	30
	Active TB	50	34 (17–62)	62
Korea	Control	10	23 (21–25)	90
	Active TB	10	51 (24–77)	40
South Africa	Suspect TB	31	32 (18–56)	26
	Active TB	20	32 (19–57)	65
Multiple^*^	Leprosy reactions	53	(18–69)	68
the Netherlands	Leprosy^†^	33	34 (18–57)	62
	Sarcoidosis	50	43 (26–57)	60
	Control	80	38 (21–67)	36
	BCG vaccinated	12	27 (23–57)	33
	Pneumonia (Leiden)	40	66 (23–93)	60
	Pneumonia (Nieuwegein)	28	73 (34–91)	57

*In the table the different cohorts are described regarding the country of origin of the samples, the disease classification, and the total number of samples per group as well as the demographic info on age and sex*.

* *Nepal, Brasil, Ethiopia*.

† *Diagnosis made in the Netherlands*.

#### Tuberculosis

Smear, GeneXpert or sputum culture positive pulmonary TB patients, LTBI patients and treated TB patients as well as endemic controls (in different combinations) were included from various demographic locations: Italy (31), the Gambia, Korea and South Africa (Table 1). Patients with active pulmonary TB disease, referred to as “TB” in the manuscript, were diagnosed based on local, routine methodology. Active pulmonary TB was sputum-culture confirmed (BACTEC™, Becton-Dickinson, USA), or based on positive Xpert Mtb/RIF assay (Cephaid Inc., Sunnyvale, CA, USA), patients were included within 7 days of TB treatment initiation. Latent TB infection, LTBI, was determined by Quantiferon TB Gold-in tube positivity (Qiagen, The Netherlands).

In the cohort from South Africa people suspected for TB were used as controls, these people were presenting with symptoms compatible with active TB but had negative X-ray and negative sputum cultures for TB. These suspected TB patients were seen again after 2 months and had recovered spontaneously or after appropriate (non-TB related) treatment. All TB patients were HIV-negative, as were the endemic controls. Additionally, from Italy both LTBI (QuantiFERON TB Gold-In-tube-positive individuals) and successfully treated TB patients (2–72 months after end of therapy) were included. TB patients from the Gambia were followed over time (1, 2, and 6 months after diagnosis), until completion of successful treatment.

#### Other mycobacterial diseases and vaccination

Leprosy patients (mostly immigrants with mixed ethnic backgrounds) at diagnosis of primary leprosy were included in the Netherlands. In addition, patients with type-1 reactions were enrolled in Brazil, Nepal and Ethiopia. Furthermore, we measured C1q in healthy Dutch individuals who were vaccinated with BCG Danish strain 1331 (Statens Serum Institut, Denmark) and followed over time (32).

#### Other pulmonary diseases

Patients with community acquired pneumonia were included in the Netherlands, one cohort comprised patients admitted to the intensive care unit in a tertiary care hospital in Leiden (one patient was HIV-infected with a normal CD4 count and one suffered from sarcoidosis) and the other cohort comprised patients admitted to a hospital ward of a non-academic teaching hospital in Nieuwegein. From both groups of pneumonia patients paired samples from the time of diagnosis and after recovery (10–124 days later) were available. Finally, samples were included prior to initiation of treatment from sarcoidosis patients in the Netherlands that had pulmonary involvement.

#### Additional control group

As a reference group we included a panel of Dutch healthy controls, not suffering from major infections or autoimmune disease.

#### Ethics statement

Blood was obtained from individuals upon signing an informed consent. All studies comply with the Helsinki declaration. The use of the samples in this study was approved by local ethical committees. For Italy, Ethical Committee of the L. Spallanzani National Institute of Infectious diseases (02/2007 and 72/2015); The Gambia (SCC1333); Korea, Institutional Review Board for the Protection of Human Subjects at YUHS; South Africa, Health Research Ethics Committee of the Faculty of Medicine and Health Sciences at Stellenbosch University (N13/05/064); Brazil, National Council of Ethics in Research and UFU Research Ethics Committee (#499/2008); Nepal, Health Research Council (NHR#751); Ethiopia, Health Research Ethical Review committee Ethiopia (NERC#RDHE/127-83/08); The Netherlands leprosy patients, (MEC-2012-589); sarcoidosis patients, Medical research Ethics Committees United of the St Antonius (#R05.08A); BCG vaccinated individuals, Leiden University Medical Center Ethics Committee (P12.087); control group, (P237/94); pneumonia Leiden (P12.147); pneumonia Nieuwegein (C-04.03 and R07.12).

### Gene expression analysis

Global transcriptomic analyses have been performed to compare patients with active TB disease with latently infected individuals. In addition, transcriptomes from patients with TB disease were compared with patients with other diseases, such as sarcoidosis, pneumonia or lung cancer (5–7, 33–37). Microarray data from these studies, publically available in Gene Expression Omnibus (GEO) (GSE37250, GSE19491, GSE39941, GSE28623, GSE73408, GSE34608, GSE42834, GSE83456), were retrieved from GEO and re-analyzed. Several of the studies described multiple independent cohorts, which we analyzed separately for each population [from Malawi (2), South Africa (3), United Kingdom (2), Kenya (only TB vs. LTBI), The Gambia (only TB vs. LTBI), USA and Germany (only TB vs. other diseases)]. All data were extracted from GEO and compared in the same way using GEO2R, thus not relying on the analysis performed in the original manuscript. GEO2R compared two or more groups of samples in order to identify genes that are differentially expressed across experimental or clinical conditions. Here, lists of differentially expressed genes between TB and LTBI or TB and other diseases (with a significance of *p* > 0.05 and a factorial change of >2 or <0.5) were generated. A list of complement genes and two reference genes was used to assess possible differential expression for each individual gene for each study population. All studies/populations with significant differential expression for a particular gene between patients with TB compared to LTBI/other diseases were enumerated and expressed as the percentage of the total number of comparisons investigated. E.g., differential expression of C1QA between TB and LTBI was observed in only 2/9 populations investigated (22%) whereas C1QB differed in 8/9 populations (88%). For all populations with significant differential expression, the factorial change (the difference between gene expression in TB patients compared to LTBI/other diseases) was calculated and plotted.

### Animals

Non-human primate (NHP) serum was available from a biobank of samples collected from earlier TB studies in healthy, purpose-bred rhesus macaques (*Macaca mulatta*) for which ethical clearance was obtained from the independent ethical authority according to Dutch law. All housing and animal care procedures were in compliance with European directive 2010/63/EU, as well as the “Standard for Humane Care and Use of Laboratory Animals by Foreign Institutions” provided by the Department of Health and Human Services of the US National Institutes of Health (NIH, identification number A5539-01). Longitudinal banked serum samples were available for C1q analysis. Animals were non-vaccinated or vaccinated with BCG [BCG Danish 1331 (Statens Serum Institute, Denmark)] (*n* = 23) and experimentally infected via bronchoscopic instillation of 500 CFU of *M*. Erdman. Prior to infection and 3, 6, 12, 24, 36, and 52 weeks post-infection samples were collected and stored. Animals were sacrificed when reaching early humane endpoints (acute progressors) or after reaching the pre-defined end-point (52 weeks; non-progressors). Broncho-alveolar lavage (BAL) samples were available from six animals infected with 1–7 CFU of M. Erdman prior to infection and 6 or 12 weeks post-infection.

### Detection of C1q by ELISA

C1q levels in sera were measured using an in-house developed ELISA. Maxisorp plates (nunc) were coated overnight with mouse anti-human C1q (2204) (38), Nephrology department, LUMC) in coating buffer (0.1 M Na_2_CO_3_, 0.1 M NaHCO_3_, pH9.6). Plates were washed and blocked with PBS/1%BSA for 1 h at 37°C. After washing, a serial dilution of a pool of normal human serum (NHS) was applied as a standard and samples were added in dilution to the plate, all in duplicate, and incubated for 1 h at 37°C. Human sera were diluted 1:8,000 and NHP sera 1:4,000, the BAL fluids were diluted 1:1. After washing, plates were incubated with rabbit anti-human C1q (Dako cat#A0136) for 1 h at 37°C and for detection a goat anti-rabbit HRP (Dako cat#P0448) was used which was also incubated for 1 h at 37°C. All washing steps were performed with PBS/1%BSA/0.05%Tween. Plates were stained using ABTS and measured at absorbance of 415 nm, the measured C1q is expressed in μg/mL as compared to a C1q standard.

### Immunohistochemical staining of lung tissue for C1q

Samples were collected at autopsy from patients with fatal TB disease (*n* = 3), fatal pneumonia patients (*n* = 4) and a control that died of vascular disease (*n* = 1) at the National Institute for Infectious Diseases, Rome, Italy under local ethical approval (72/2015). Paraffin sections of 4 μm thickness were subjected to heat-induced antigen retrieval using tris/EDTA (pH 9.0) at 96°C for 30 min, and then stained with rabbit anti-human C1q (1:1,000; Dako cat#A0136) in PBS/1%BSA for 1 h at room temperature, followed by an anti-rabbit Envision (Dako) HRP conjugated antibody also for 1 h at room temperature, with DAB+ as the chromogen. Negative control rabbit immunoglobulin fraction (Dako) was used as a negative control in the same concentration as the primary antibody. Sections were counterstained with Haematoxylin (Klinipath; 4085.9001).

### Statistics

Statistical analyses were carried out using SPSS statistics version 23 (IBM) or Graphpad Prism version 7. To compare C1q levels the Mann-Whitney U test, Kruskall-Wallis and Dunn's multiple comparisons test were used. In all graphs the median is shown unless indicated otherwise. Receiver operating characteristic (ROC) analysis was performed to assess the sensitivity and specificity of C1q as biomarker and was expressed as Area Under the Curve (AUC).

## Results

### *C1Q* expression is upregulated in patients with active TB disease

**Figure 1:**
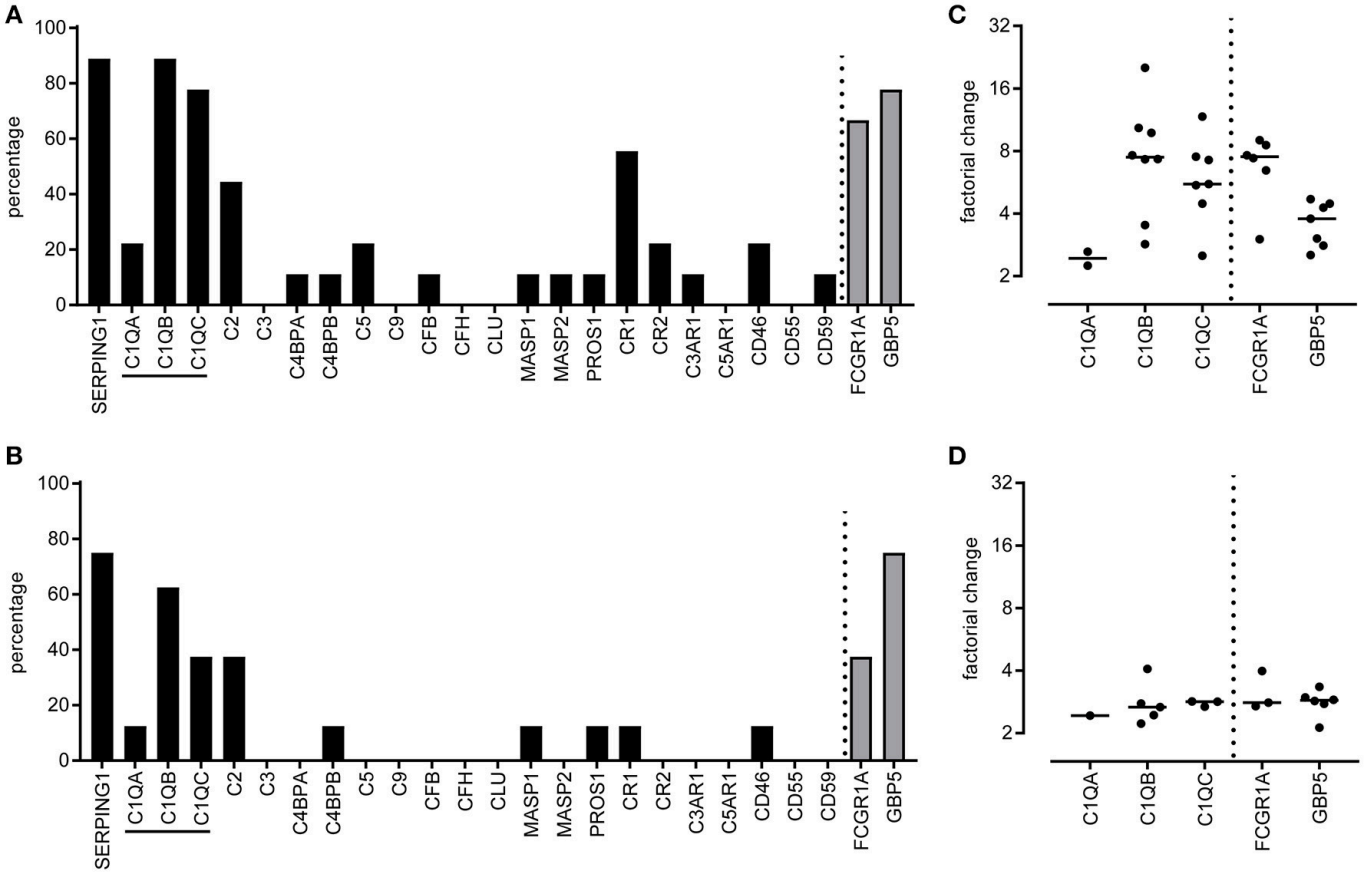
Differentially expressed complement genes in whole blood tuberculosis transcript signatures. Tuberculosis specific transcript signatures, from various populations, were investigated for the presence of differentially expressed complement genes using a tuberculosis RNA biomarker database. Publically available transcriptome data was retrieved from Gene Expression Omnibus (5–7, 33–37) and analyzed using GEO2R. Data were available for nine populations comparing active TB with LTBI and for 8 populations comparing active TB with other diseases. For each population we determined if the complement family genes were differentially expressed between TB and LTBI or other diseases. Differential expression was defined as an adjusted *p*-value <0.05 and more than 2-fold change. Differential expression of a gene between TB and LTBI or other diseases was expressed as percentage of the total number of populations investigated. The differential expression of complement genes was scored **(A,B)** as well as the mean factorial change for the C1q genes, *C1QA, C1QB*, and *C1QC* **(C,D)** for the comparisons TB vs. LTBI **(A,C)** and TB vs. other diseases **(B,D)**. As a reference two other highly upregulated potential diagnostic TB markers, *FCGR1A*, and *GBP5*, were included in the analyses.

Publically available microarray data from TB patients were retrieved from Gene Expression Omnibus, all data were ranked as differentially expressed between TB patients and either LTBI or other diseases (5, 7, 33–37). These studies contained information from diverse populations (Malawi, South Africa, United Kingdom, Kenya, The Gambia, USA, and Germany). A list of complement gene expression patterns was generated and the number of microarray studies that reported differential complement gene expression between patients with TB and LTBI (Figure 1A) or other diseases (Figure 1B) was enumerated. Complement genes *C1QB, SERPING1* were expressed at higher level (more than 2-fold) in 8/9 (88%) of studies comparing patients with active TB to LTBI (Figure 1A). C1QC was expressed at a higher level in TB patients in 7/9 studies (78%), whereas C1QA expression was only increased in TB patients in 2/9 studies (22%; Figure 1A). The observed factorial changes in the expression of these complement genes between TB patients and LTBI individuals were comparable with the changes as seen for *FCGR1A* and *GBP5*, which were previously described as promising and highly consistent biomarkers of active TB (Figure 1C). A similar pattern, although less pronounced, was seen when comparing TB patients with patients having another lung-disease (Figures 1B,D). As C1q is abundantly present, easy to measure and stable, therefore we continued to analyse C1q protein levels.

### C1q is significantly increased in serum of TB patients

**Figure 2:**
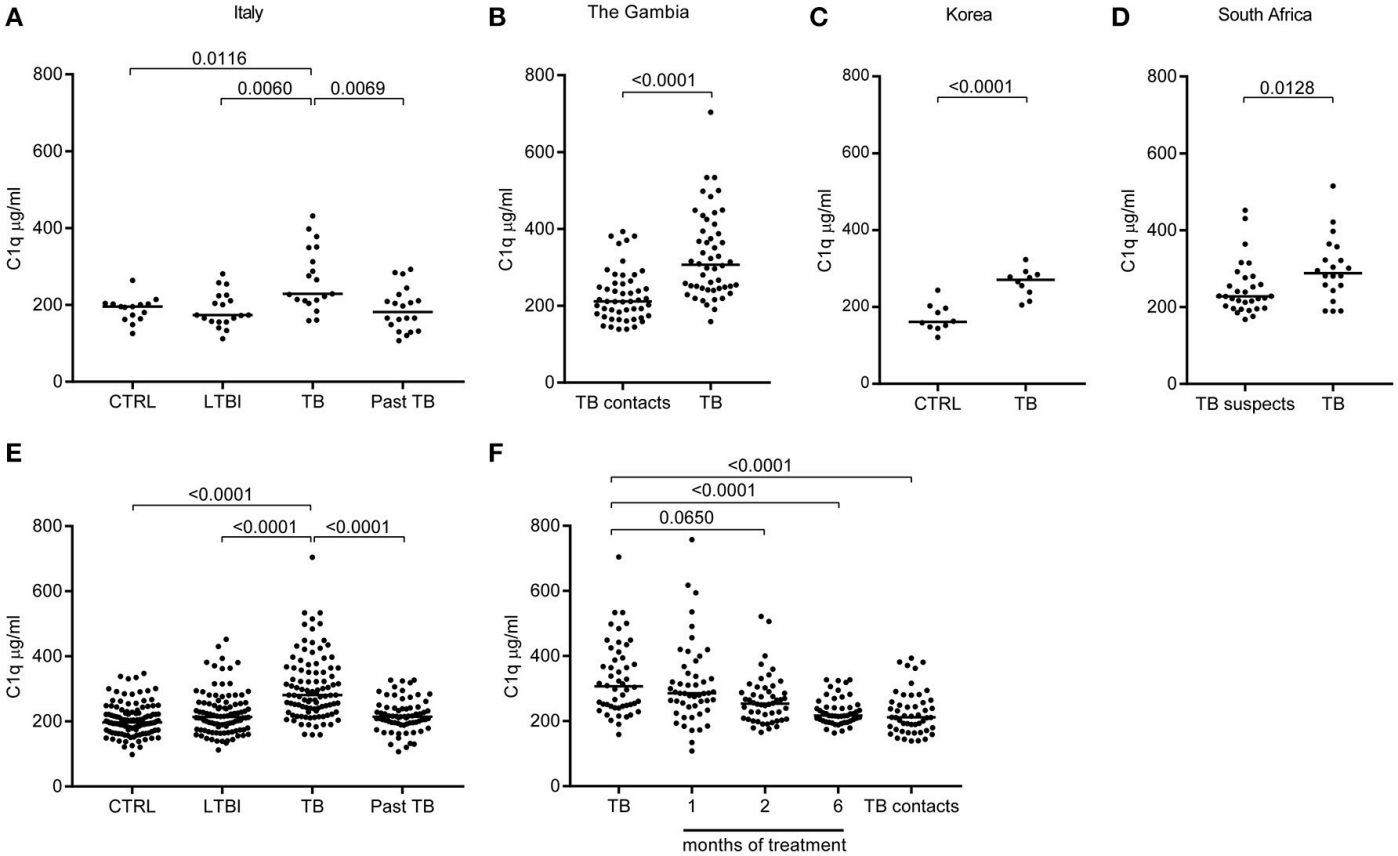
C1q serum levels are increased in patients with pulmonary Tuberculosis. C1q levels (μg/ml) were measured with ELISA in sera from TB patients (active disease) and controls from different cohorts. First the results from the independent and geographically different cohorts are depicted. TB patients from Italy were compared to Latent TB infected (LTBI), to Past TB (patients that were successfully treated for TB) and to endemic controls **(A)**. TB patients from The Gambia were compared to TB contacts **(B)**. TB patients from Korea were compared to endemic controls **(C)**. From South Africa the TB patients were compared to patients suspected for TB but confirmed non-TB **(D)**. Subsequently, data from the cohorts were pooled: control (CTRL) comprises Dutch healthy controls, combined with the CTRL from Italy and Korea; moreover, healthy individuals prior to vaccination with BCG were included (*n* = 117). Latent TB infected (LTBI) comprises LTBI from Italy, TB suspects (confirmed non-TB) from South Africa and TB contacts from the Gambia (*n* = 100). Active tuberculosis (TB) from Italy, the Gambia, Korea and South Africa (*n* = 99); Past TB are patients that were successfully treated for TB and combined the past TB from Italy and the Gambia samples after 6 months of treatment (*n* = 71) **(E)**. From the Gambia the TB patients were followed over time during treatment, TB contacts are shown on the right (*n* = 50) **(F)**. Results were analyzed using the Mann-Whitney *U* test, >2 groups Kruskall-Wallis and Dunn's multiple comparisons test. The treatment months were compared to TB diagnosis with Friedman Test and Dunn's multiple comparisons test.

C1q protein levels were measured in sera from TB patients and controls from independent and geographically distinct cohorts (Figures 2A–D). The levels of C1q were significantly higher in sera from patients with active TB as compared to their respective controls in all cohorts: Italy (Figure 2A), The Gambia (Figure 2B), Korea (Figure 2C) and South Africa (Figure 2D). LTBI individuals and successfully treated TB patients had serum C1q levels similar to controls (Figure 2A). Combined analysis of all TB patients, control groups, LTBI and the successfully treated TB patients from the different cohorts revealed that serum C1q protein levels are significantly (*p* < 0.001) increased in active TB (Figure 2E).

The Gambian TB patients were followed over time which allowed us to investigate C1q levels during successful treatment. At 1 month of treatment the median serum C1q level was still increased, however, the level of C1q began to decrease after 2 months (*p* = 0.0650), resulting in complete normalization compared to the TB contacts after 6 months of successful treatment (*p* < 0.001; Figure 2F). Thus, serum C1q protein levels were significantly elevated in patients with active TB, and levels decreased to the level of the control population during successful treatment. This further indicates that increased C1q levels are associated with active TB disease and do not reflect genetic variation in C1q expression.

### Vaccination with BCG does not increase C1q levels in serum

**Figure 3:**
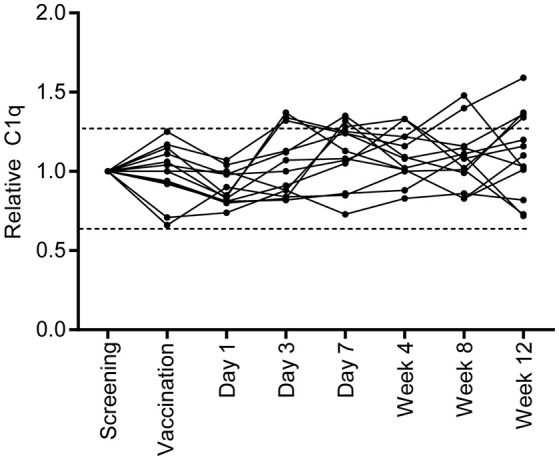
BCG vaccination does not induce a similar C1q upregulation. Healthy individuals who were vaccinated with BCG (*n* = 13), were followed over time and C1q serum levels were measured. C1q levels were first calculated to μg/ml and for each individual set to 1 using the measurement of the C1q level before vaccination. The dotted lines indicate the variation that is present in C1q levels at the time of screening and prior to vaccination.

To investigate if vaccination with *M. bovis* BCG, a live replicating mycobacterium, induced a similar increase in serum C1q levels, samples were taken before and after BCG vaccination of healthy Dutch volunteers and C1q levels were measured (Figure 3). Samples taken at screening and directly before vaccination showed minimal variation in C1q levels, reflecting normal variation within individuals. BCG vaccination did not induce fluctuations in C1q levels larger than this naturally observed variation. Thus, BCG vaccination did not increase C1q levels in contrast to what was observed in TB disease, despite the presence of live, replicating mycobacteria.

### C1q levels are increased in active TB compared to other diseases

**Figure 4:**
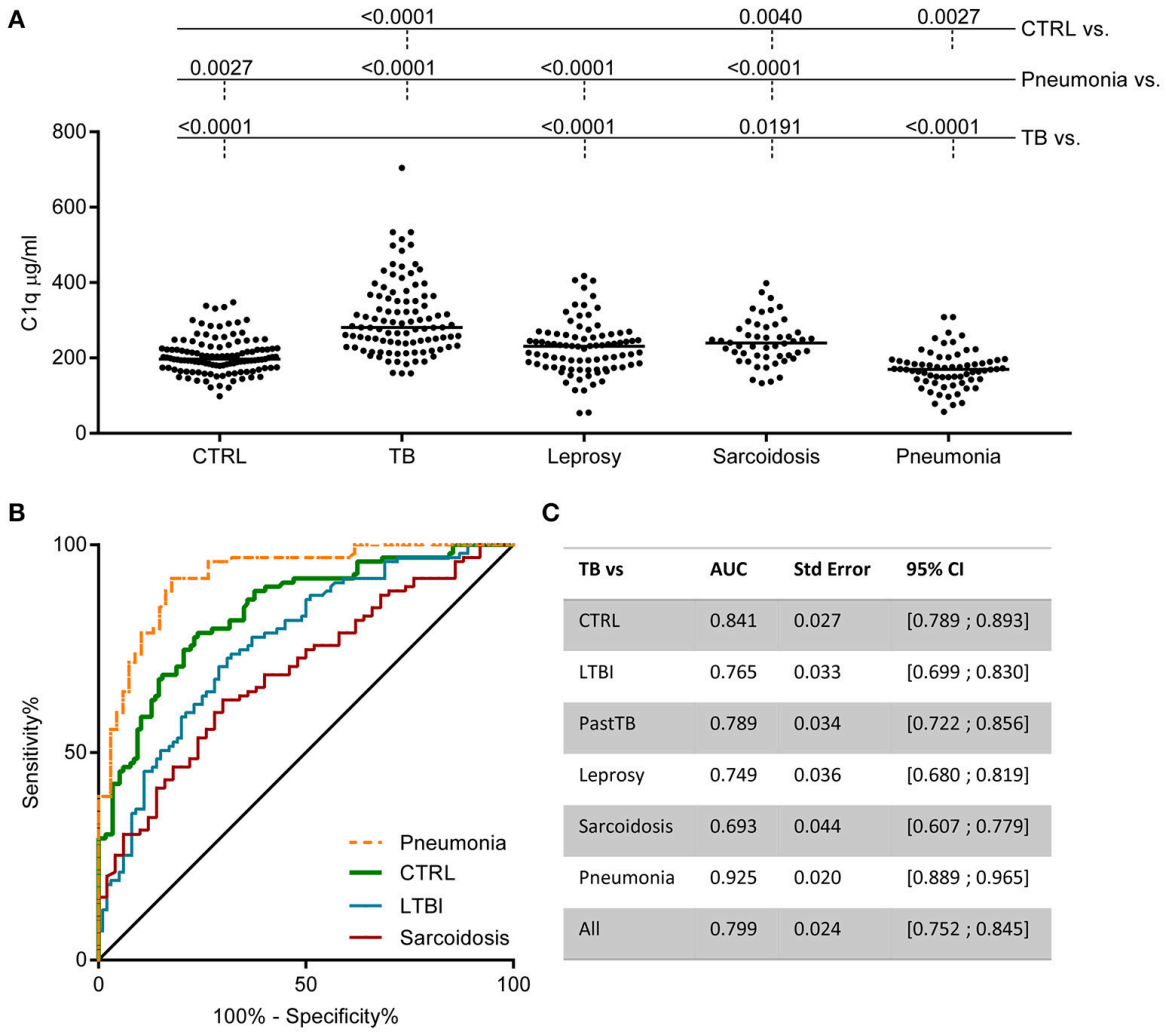
Increased C1q serum levels are associated with TB. C1q was measured in various cohorts by ELISA. The pooled data from Figure 2E control population (CTRL) and TB (active disease) is now compared to other diseases: leprosy (*n* = 86), sarcoidosis (*n* = 50), and community acquired pneumonia (*n* = 68) **(A)**. Differences between groups were analyzed using Kruskall-Wallis and Dunn's multiple comparisons test. Both the leprosy and the pneumonia cohort comprise two patient groups. The data for these individual groups is visualized in Supplementary Figure E1. ROC analysis of the ability of C1q to distinguish TB from CTRL, LTBI, sarcoidosis, and pneumonia are plotted together **(B)** and for all comparisons the Area Under the Curve (AUC) was calculated and summarized in the table **(C)**.

To investigate the specificity of increased C1q levels for active TB, sera from patients with other diseases with similar symptoms and radiological abnormalities (pneumonia, sarcoidosis) and sera from patients with other mycobacterial disease [leprosy, primary disease or type 1 (acute pro-inflammatory) reactions] were analyzed. C1q levels in sera from patients with TB were significantly higher compared to sera from patients with leprosy, pneumonia or sarcoidosis (Figure 4A, data from TB patients and controls same as used in Figure 2E). Some individual leprosy patients had C1q protein levels above the median of TB patients but this was not related to either primary disease or having type I reactions (Supplementary Figure E1A). Patients with sarcoidosis showed a slight increase in C1q levels compared to the controls. In contrast, patients with community acquired pneumonia showed a significant decrease in C1q levels compared to the control population. The reduced levels observed in patients with pneumonia at diagnosis was associated with the disease state since samples included from the same individuals at later time points had normal C1q levels (Supplementary Figures E1B,C).

To assess the value of C1q as possible TB biomarker, the sensitivity, specificity and the positive likelihood ratio (LR+) were calculated from C1q concentration cut-offs (10). With a cut-off at the 95th percentile of the control population (300.2 μg/ml C1q) the sensitivity is 42% with a specificity of 91% resulting in a LR+ of 4.96. Application of a cut-off at the maximum of the control population (347.6 μg/ml) resulted in a sensitivity of 29% and a specificity of 97% resulting in a LR+ of 8.99. The capacity of serum C1q to discriminate active TB from LTBI, pneumonia and sarcoidosis was also analyzed using ROC analyses and expressed as AUC (Figures 4B,C). The AUC of C1q levels for TB vs. LTBI was 0.77, for TB vs. sarcoidosis 0.69, and for TB vs. pneumonia even an AUC of 0.93 was achieved.

### C1q is locally present in the lungs of TB patients

**Figure 5:**
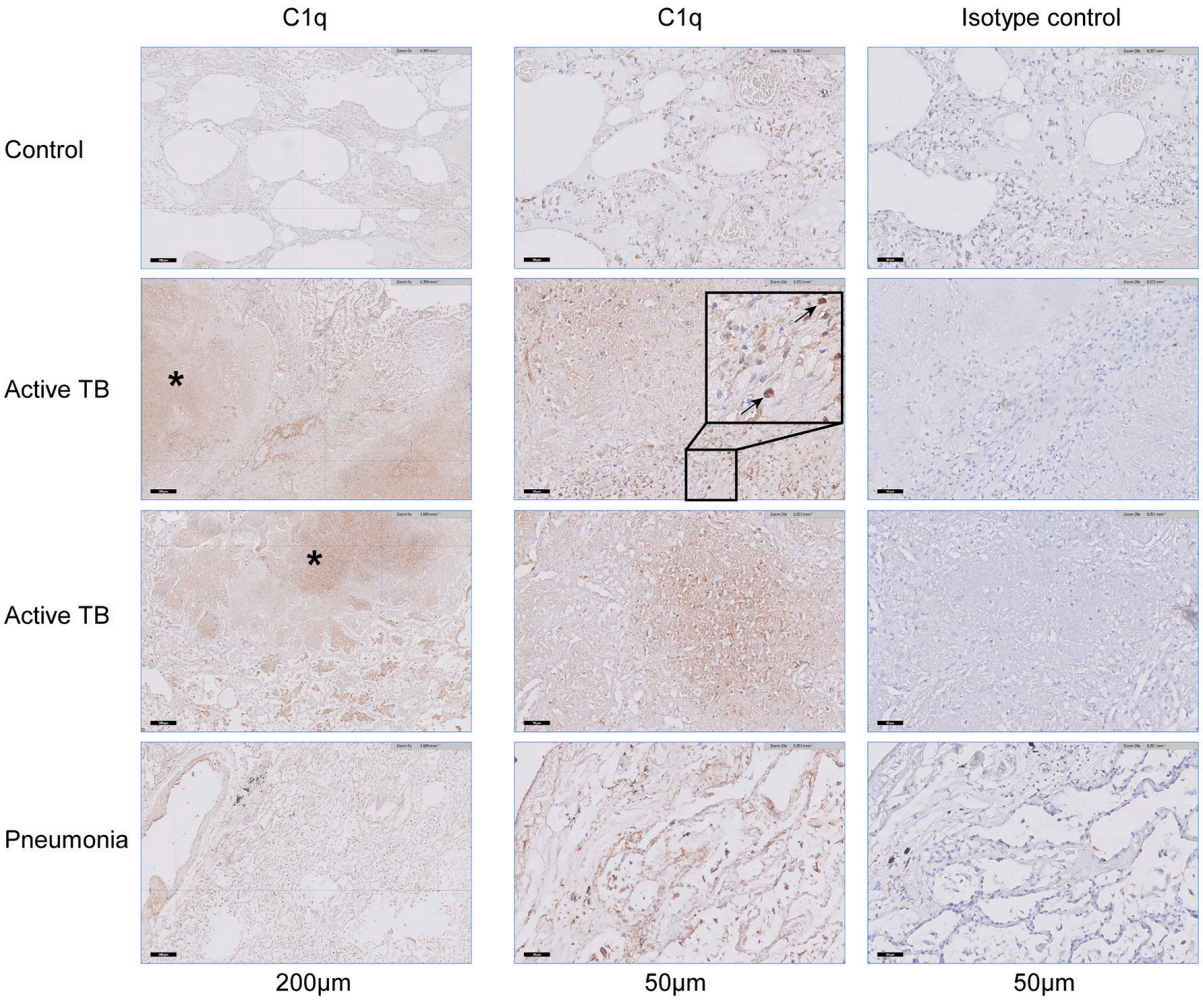
C1q accumulates in lung tissue of patients with fatal pulmonary TB. Lung tissue, obtained at autopsy from a non-pulmonary disease control (*n* = 1), fatal active TB patients (*n* = 3), or patients with lethal pneumonia (*n* = 3) were stained for the presence of C1q. The left column shows the presence of C1q in a section of the control, two active TB patients and a pneumonia patient, scale bar at 200 μm. The middle column shows the same samples stained for C1q, while the right column shows consecutive tissue slides stained with a matched control antibody, scale bars at 50 μm. Necrotic areas that stain positive for C1q are highlighted with an asterisk (*) and individual cells that stain positive for C1q are highlighted with arrows.

So far circulating RNA and protein levels of C1q have been analyzed, which reflect the systemic response to TB. Additionally, we analyzed the local C1q production or deposition in response to *M.tb* infection by staining lung tissue. Staining lung tissue from a control revealed scarce C1q staining with only few C1q positive macrophage-like cells in the lung parenchyma and in the intra-alveolar space (Figure 5). In contrast, lung tissue of fatal TB patients revealed, next to the intra-alveolar C1q positive cells also a pronounced C1q staining both in the necrotic centers of the granulomas and in the surrounding lung tissue with predominantly macrophage-like cells staining positive. Lung-tissue from patients that succumbed from pneumonia showed C1q staining predominantly in the intra-alveolar space. Staining of consecutive sections with an isotype control did not reveal any staining, also not in the necrotic centers, confirming specific staining for C1q. Thus, C1q protein is locally detected at an increased level in the lungs of TB patients (*n* = 3) compared to tissue samples from a non-pulmonary disease control (*n* = 1) or pneumonia patients (*n* = 4).

### Non-human primates with active TB disease also display increased serum C1q levels

**Figure 6:**
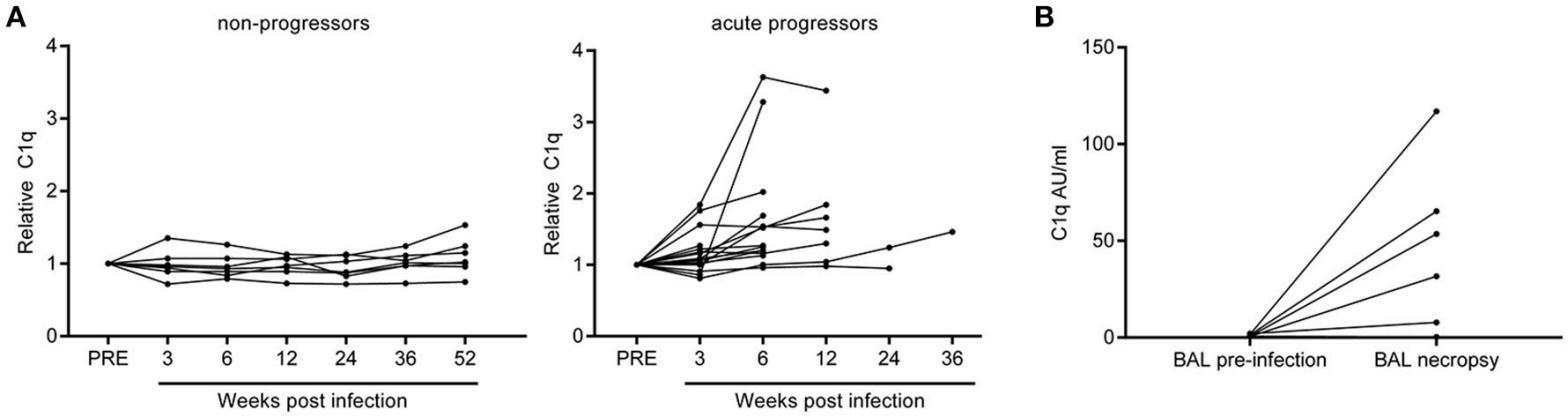
Rhesus macaques infected with *Mycobacterium tuberculosis* display increased levels of C1q in serum and broncho-alveolar lavage fluid. C1q levels were measured in serum samples from rhesus macaques (*n* = 23) which were infected with *Mycobacterium tuberculosis* and followed over time. C1q levels were first calculated as Arbitrary Units (AU) per ml after which the C1q level of each animal at baseline was set as 1. Animals which controlled the infection are termed non-progressors and animals reaching a premature humane endpoint due to active disease progression are termed acute progressors **(A)**. Separately, C1q was measured in broncho-alveolar lavage (BAL) fluid obtained from animals before and 6 or 12 weeks after infection with *M.tb* (*n* = 6) and calculated as AU/ml **(B)**.

Non-human primate (NHP) *M.tb* infection models are widely used to study pathogen-host interactions and for pre-clinical evaluation of vaccine candidates (39). After infection, rhesus macaques develop TB disease which closely resembles human TB in most aspects. Sera banked over the course of a long-term follow-up study in rhesus macaques were used to determine C1q levels after experimental *M.tb* infection. 14 Out of 16 animals with active progressive disease, that had reached an early humane endpoint due to exacerbation of TB disease, had increased C1q levels compared to their baseline C1q levels before infection (Figure 6A). Such a rise in C1q levels was absent in six out of seven animals that did not develop overt disease, but controlled the infection over an extended period of time up to 1 year post-infection (Figure 6A). Additionally, in an separate cohort of *M.tb* infected NHPs we detected elevated C1q levels in five out of six broncho-alveolar lavage (BAL) fluid samples taken before necropsy, while no C1q could be detected in paired BAL fluids taken prior to infection (Figure 6B). The observed differences could not be explained by any differences in BAL volume recovery and thus these data reflect a true local increase in C1q after *M.tb* infection.

## Discussion

The accurate and fast identification of patients with active TB remains challenging, largely because of limitations in the current diagnostic tools to differentiate active TB from other diseases, as well as LTBI. Extensive searches for biomarkers that can discriminate active TB from other diseases with similar clinical presentation as well as from LTBI have been recently reported (3, 40). Several studies reported genes encoding for complement components to be upregulated in TB (15–19). Here, we compiled available genome wide gene expression data for TB compared to LTBI and TB compared to other lung diseases (5–7, 33–37) and observed that in particular C1q encoding genes were highly upregulated. Interestingly, these observations were made in studies using RNA from whole blood, indicating increased transcription of C1q genes in circulating blood cells, most likely monocytes/macrophages. Since C1q is not a typical acute-phase protein we were interested to confirm and validate these findings at the protein level. We have therefore measured C1q protein levels in serum and confirmed increased levels in patients with active TB, but not in other diseases with a similar clinical presentation, such as pneumonia or sarcoidosis, or mycobacterial exposure. C1q protein is also present in the lung tissue of deceased TB patients. The increased levels of local and circulating C1q in TB were replicated independently in a NHP TB infection model.

Literature suggests that expression of the three C1q genes *C1QA, C1QB*, and *C1QC* is regulated in a similar manner (41). However, upon IFNγ-stimulation upregulation of *C1QB* is higher than *C1QC*, which is higher than *C1QA* (41). Similarly, our data also showed upregulated expression of *C1QB* and *C1QC* genes and *C1QA* was less frequently observed in active TB, also in the analysis from Cai et al. the extend of increase in the expression of *C1QA* was less pronounced as compared to the increase in expression of *C1QB* and *C1QC*. Since C1q protein production requires equal ratios of all three chains, the detected increase in C1q protein levels in TB patients indicates that all chains are expressed. The low detection of *C1QA* may thus be technical and reflect a poor capture of *C1QA* expression on the microarrays in general.

We measured C1q protein levels in four different geographical cohorts from Italy, The Gambia, Korea and South Africa. C1q levels were increased in patients with active TB compared to all relevant control populations. Importantly, treatment normalized serum C1q levels to those of endemic controls, indicating that the upregulation of C1q was associated with the disease and not intrinsic to the individuals. BCG vaccination, although being a live replicating mycobacterial vaccine, did not induce increased C1q levels. Although leucocytes of patients with leprosy reactions were reported to express increased levels of *C1QA, B*, and *C* (42), the cohorts analyzed here did not universal show increased serum C1q levels. Individual patients might have somewhat increased levels, both in this study (type 1 reaction) and a previous report (type 2 reaction) (43), which warrants more detailed analyses. We speculate that the different pathophysiologies of TB and leprosy, in particular the different levels of systemic inflammation and immune activation which are generally higher in active TB than in leprosy are responsible for the difference in C1q levels between the two diseases, even though both are caused by pathogenic mycobacteria.

To further evaluate the potential of C1q as a biomarker for active TB, C1q levels were compared to those of disease relevant controls as patients with untreated pulmonary sarcoidosis or pneumonia. Patients with TB had significantly higher circulating C1q levels as compared to patients with sarcoidosis or patients with pneumonia. Thus, the upregulation of C1q likely does not reflect a general response to inflammation. Patients with pneumonia rather had decreased C1q levels compared to controls. Treatment of pneumonia normalized the levels of circulating C1q, suggesting that the observed decrease in C1q is non-genetic and likely associated with the disease process. ROC analysis indicated that C1q, even as a single marker, readily discriminated active TB from all other diseases investigated here. Furthermore, also in a setting of an experimental NHP model of TB disease we observed in both sera and BAL fluid increased C1q levels in animals with symptomatic TB disease compared to the level prior to infection with *M.tb*. This was not seen in animals that did not progress to TB disease, suggestion again an association with TB disease rather than infection only. These data fully agree with and support the observations described above in the human cohorts.

Longitudinal follow up of TB patients during treatment revealed that circulating C1q levels normalized to the level of endemic controls. However, here protein levels did not completely normalize until the 6 months time point. Previously published data showed a rapid decrease in C1q mRNA expression levels following treatment (16, 19). Cliff et al. show that already after 1 week of treatment the blood C1q gene expression decreased (16). Cai et al. reported a significant decrease in the expression of the C1q genes at 3 months of anti-TB chemotherapy whereas they also described a reduction in C1qc protein levels after 6 months (19). Our data presented here substantially expand the number of populations, including populations from different regions of the world. Transcriptomic analysis of genes encoding complement proteins were now assessed in 9 independent populations from different TB endemic as well as non-endemic regions, strongly supporting an increase in expression of C1Q as well as SERPING1 during active TB disease in cells present in peripheral blood. Moreover, 4 independent cohorts as well as the data obtained in the NHP model show increased circulating C1q plasma levels during active TB disease, but not infection. In addition to conforming the data previously published for a Chinese population (Cai et al.) in 4 different TB cohorts, we also showed the specificity for TB disease in comparison to clinically important differential diagnoses, such as sarcoidosis and pneumonia. As C1q is produced by cells of monocytic origin, it reflects another component of the immune space compared to most currently applied TB biomarkers, such as C-reactive protein and IP-10 (44–47). In addition, C1q levels are technically easy to measure and the C1q protein is not sensitive to degradation. Therefore, we hypothesize that addition of C1q to current biomarker panels or platforms, will have additive value in discrimination of TB patients.

Low levels of C1q have been reported in several inflammatory and autoimmune diseases, such as Systemic Lupus Erythematosus. This is largely the result of C1q consumption because of immune complex mediated disease and in some rare cases caused by genetic C1q deficiency (26). However, the increased levels of C1q, as occur in TB, are observed very rarely. So far the only other clinical condition in which increased levels of C1q have been reported is Kala Azar (48). The mechanism behind the increased C1q levels observed in TB patients, or the possible functional consequences for the host are unknown, and will need further investigation. The availability of the NHP model of tuberculosis for C1q research, as demonstrated for the first time in this study, should greatly accelerate and facilitate such work.

In conclusion, we show here that circulating C1q expression is increased in 9 different populations with TB disease, moreover, elevated C1q plasma levels were observed in 4 cohorts of TB patients compared to LTBI or endemic controls. Specifically, C1q levels in TB patients were significantly increased compared clinically relevant diseases, such as sarcoidosis, leprosy and pneumonia. Moreover, we show that increased C1q levels decreased to the level of the control population during successful treatment. In analogy with human TB, C1q also validated as a biomarker of TB disease in rhesus macaques, in both serum and BAL. Increased C1q levels were only observed in animals that progressed to active disease and not in those that controlled the infection, suggesting a direct association with disease rather than with infection. Therefore, we propose that the addition of C1q measurements to current biomarker panels may provide added value in the diagnosis of active TB.

## Author contributions

RL, FV, AG, TO, SJ, and LT designed the study. RL, RdP, KD, IB, and KG performed analyses. JS, DG, CvM, MV, SV, WB, LP, FD, GW, and GG oversaw recruitment and collection of specimens. RL, SJ, and LT interpreted the data. All authors critically revised and approved the manuscript.

### Conflict of interest statement

The authors declare that the research was conducted in the absence of any commercial or financial relationships that could be construed as a potential conflict of interest.

## Supplementary material

Supplementary Figure E1 C1q levels in leprosy and community acquired pneumonia. From leprosy two different cohorts were measured, one consists out of patients that were included in the Netherlands at the moment of diagnosis, the other out of sera samples from patients included at the moment they presented with a leprosy reaction. Reference C1q levels both the control groups as the pooled data from the active TB patients are depicted from **Figure 2E (A)**. For the community acquired pneumonia cohorts, samples were available from the moment the patientswere included and a follow up sample after recovery from both in Leiden **(B)** and in Nieuwegein **(C)**.
