# Supplementary figures and images for: Complement Component C1q as Serum Biomarker to Detect Active Tuberculosis
Source: Front Immunol. 2018 Oct 23;9:2427. doi: 10.3389/fimmu.2018.02427 (PMC6206241; doi:10.3389/fimmu.2018.02427)

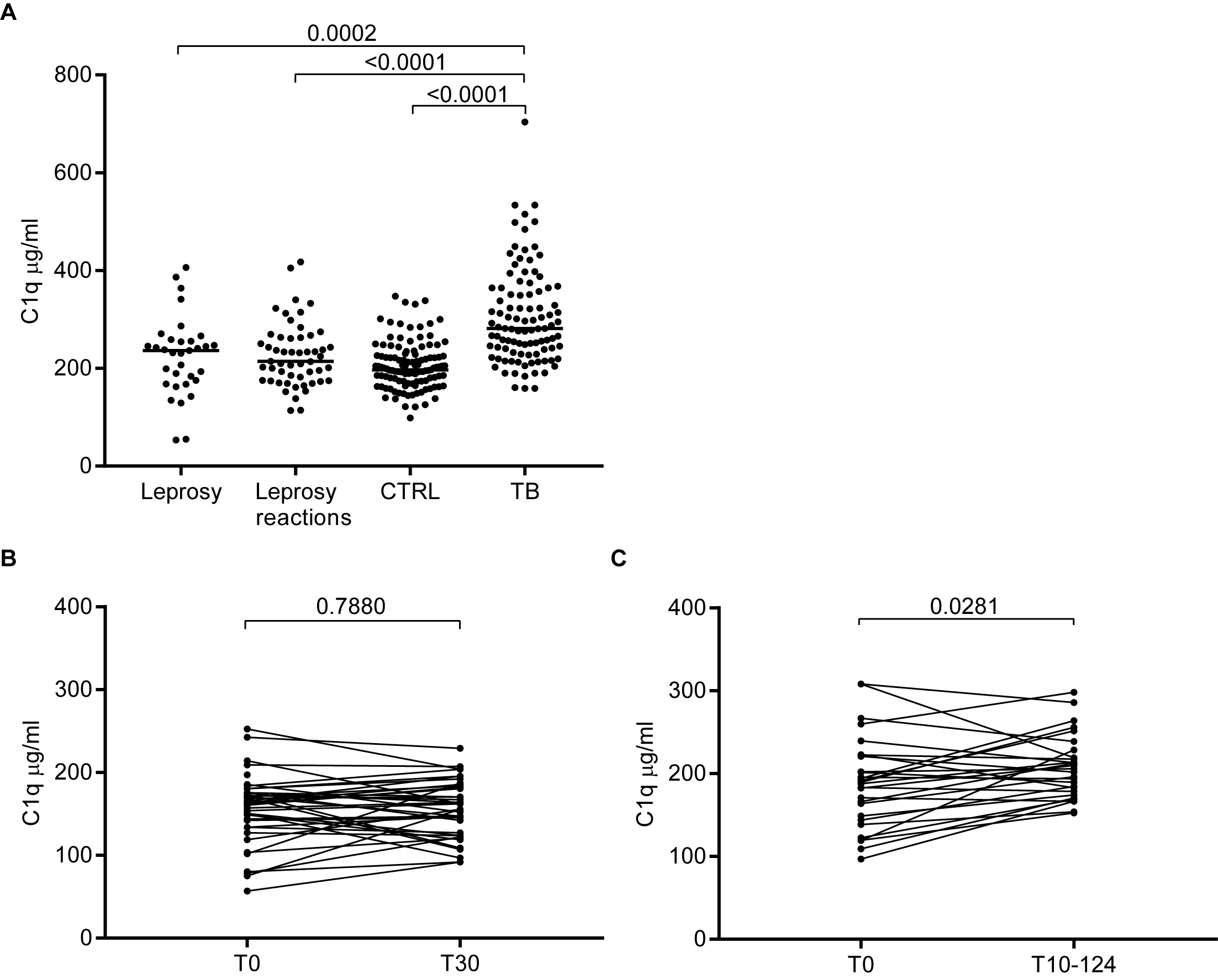

Supplement: Supplementary Figure E1 — C1q levels in leprosy and community acquired pneumonia. From leprosy two different cohorts were measured, one consists out of patients that were included in the Netherlands at the moment of diagnosis, the other out of sera samples from patients included at the moment they presented with a leprosy reaction. Reference C1q levels both the control groups as the pooled data from the active TB patients are depicted from Figure 2E (A). For the community acquired pneumonia cohorts, samples were available from the moment the patientswere included and a follow up sample after recovery from both in Leiden (B) and in Nieuwegein (C). [file Image_1.TIF]
